# The Early Impact of the People-centred Integrated Care on the Hypertension Management in Shenzhen

**DOI:** 10.5334/ijic.6539

**Published:** 2023-02-06

**Authors:** Feiyan Liu, Fangchao Liu, Jinchun Lin, Jian Wang, Jichun Chen, Jianxin Li, Jianfeng Huang, Dongsheng Hu, Xiangfeng Lu, Xizhuo Sun, Dongfeng Gu

**Affiliations:** 1 Department of Epidemiology and Biostatistics, Centre for Global Health, School of Public Health, Nanjing Medical University, Nanjing, China; 2 Shenzhen Key Laboratory of Cardiovascular Health and Precision Medicine, Southern University of Science and Technology, Shenzhen, China; 3 Department of Epidemiology, Fuwai Hospital, National Centre for Cardiovascular Diseases, Chinese Academy of Medical Sciences and Peking Union Medical College, Beijing, China; 4 Shenzhen Luohu Hospital Group, Shenzhen, China; 5 Department of Biostatistics and Epidemiology, School of Public Health, Shenzhen University Health Science Centre, Shenzhen, China; 6 School of Public Health and Emergency Management, Southern University of Science and Technology, Shenzhen, China

**Keywords:** people-centred integrated care, Luohu model, hypertension management, pharmaceutical expenditure, KAP, satisfaction

## Abstract

**Objective:**

To evaluate the impact of the integrated care in Luohu, China on the hypertension management.

**Methods:**

Hypertensive patients aged 35–74 years were recruited by the cluster-randomized sampling method from Luohu district which adopted integrated care and another district that remained original routine care during October 2018~January 2020, with 1353 and 583 patients from integrated and routine care communities, respectively. Health information, knowledge, attitude, and practice (KAP) towards cardiovascular diseases, pharmaceutical expenditure on hypertension and its comorbidities, and healthcare-related satisfaction were collected by questionnaires, with the expenditure additionally verified by hospitals’ billing records database. Continuous and categorical variables were compared by Wilcoxon test and Chi-square test, respectively. The age-standardized hypertension control rate was calculated by direct standardization.

**Results:**

The standardized hypertension management rate in the integrated care communities (45.75%) was significantly higher than that in routine care communities (14.07%) (*P* < 0.0001), while the age-standardized hypertension control rates were similar (integrated care: 50.3%, routine care: 52.65%, *P* = 0.518). The pharmaceutical expenditure on hypertension and its comorbidities in the integrated care communities was Ұ264.23 ± 357.38/month/person, lower than that in the routine care communities (Ұ354.56 ± 430.59/month/person). Patients in the integrated care had higher KAP scores (73.48 ± 11.54), compared with routine care (68.89 ± 15.51) (*P* < 0.0001). Moreover, the integrated care communities had higher satisfaction rates towards the convenience of dual referral (90.15% vs. 77.99%) and service quality (95.18% vs. 87.81%) than routine care communities (*P* < 0.0001).

**Conclusion:**

The practice of the integrated care in Luohu has substantially improved the hypertension management and the healthcare-related satisfaction while with relatively low pharmaceutical expenditure. The investigation of long-term impact of the integrated care on hypertension control and management is warranted.

## Introduction

Chronic diseases remain the leading cause of premature mortality and disease burden, especially in China [1]. In order to control the epidemic of chronic diseases, limited community- or workplace-based control programs on hypertension and diabetes had been conducted in China before the early of 1990s [2,3,4,5]. In 1997, Chinese government proposed a pilot plan of community-level comprehensive prevention and treatment of chronic non-communicable diseases [6]. Since 2009 the beginning of China’s health reform and afterwards the nationwide setting up demonstration areas for comprehensive prevention and treatment of chronic diseases in 2010, the low capacity of primary healthcare and health inequities faced by patients with chronic diseases have been gradually improved [7,8,9]. However, the medical system in China until the most recently has been hospital-centred and treatment-oriented, accompanied by the problems of fragmentation of medical services, service quality gap in inter-facilities, and uneven healthcare efficiency in different regions [10].

People-centred integrated care is now considered to be the most effective way to address issues such as epidemiological shifts, population aging, and increased demands for long-term health care [11]. Over the past decade, high-income countries have been exploring and evaluating integrated models of healthcare [12]. In low- and middle-income countries, the integration of organization, finance, and service are difficult due to the lack of strong policy support and massive investment, and little information is available on the effectiveness of integrated care in these countries [12,13,14]. To prevent and control chronic diseases, especially hypertension and diabetes, an integrated care model was launched in Luohu district, Shenzhen city in August 2015, known as the Luohu model. The Luohu model innovated the hospital management system, and adopted a series of measures to strengthen the service capability of primary healthcare and developed a medical insurance payment reform oriented with health outcomes. Unlike integrated care models in other middle-income countries which focused on improving investing and redesigning priority services, Luohu model is an important transformation of health-care systems from hospital-centred and treatment-focused to people-centred and community-based integrated health-care system by strengthening primary care and the service capacity of community health centres (CHCs), realizing the integration of organizational structures, finances, information system, and multiple types of health service [12]. The Luohu model is a practice of an integrated care in Shenzhen, China. The evaluation of the integrated care in Luohu is helpful to clarify the implication for the application in other places in China.

Previous studies qualitatively evaluated the background, actors, contents, and process of the establishment of the Luohu model, and concluded that the Luohu Hospital Group transformed the fragmented healthcare system into a tightly integrated care delivery [15,16,17]. This study will address the impact of the early practice of the integrated care model in Luohu on chronic disease management, especially for hypertension, which is a major risk factor of cardiovascular disease [18,19].

## Methods

### Study Population

**Figure 1:**
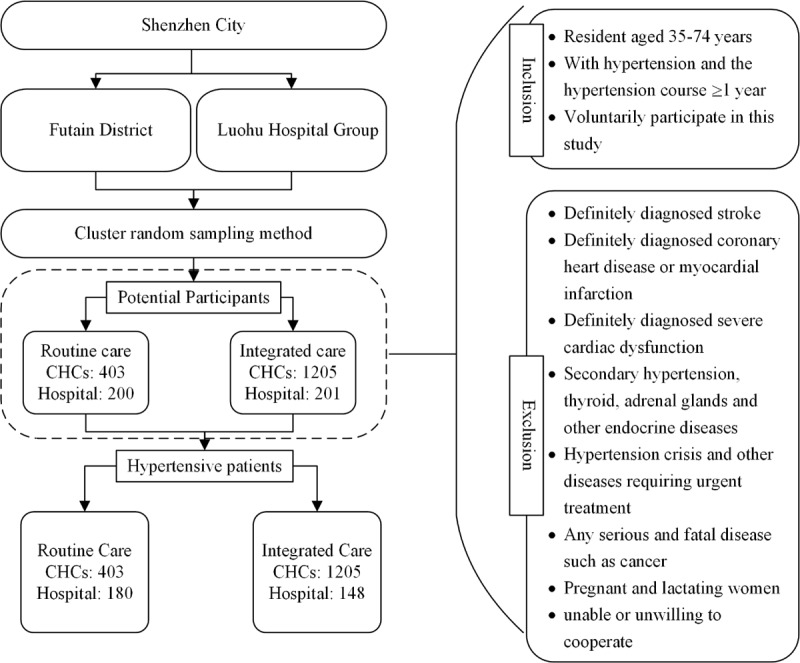
Flow chart of the recruitment plan for hypertensive patients in routine care communities and integrated care communities. *Note*: CHC: community health centre.

This is a community study performed from October 2018 to January 2020. Participants with hypertension were recruited from CHCs and hospitals’ out-patient and in-patient departments in Luohu and Futian districts through a cluster randomized sampling method. All eligible participants were aged 35–74 years, who were residents inhabited for 6 months or more in Shenzhen. They were diagnosed with hypertension by doctors or with systolic blood pressure (SBP) ≥140 mmHg and/or diastolic blood pressure (DBP) ≥90 mmHg or taking antihypertensive medication [20]. All participants had been diagnosed with hypertension for at least 1 year by the time of the survey. Individuals with stroke, coronary heart disease, myocardial infarction, severe cardiac dysfunction, liver and kidney dysfunction, tumors, and other severe diseases were excluded. After initial screening, a total of 2009 potential participants were assessed for eligibility (Figure 1). Potential participants from hospitals were recruited in a 1:1 ratio between Luohu and Futian districts, with 201 individuals in Luohu district and 200 in Futian district. Potential participants from CHCs in Luohu and Futian district were recruited in a ratio of 3, to 1, with 1205 individuals in Luohu district and 403 in Futian district. Seventy-three individuals who did not meet the inclusion criteria, were excluded. Finally, 1936 participants with hypertension were recruited in the current study, including 1353 participants in the integrated care communities (CHCs: n = 1205, hospital: n = 148) and 583 in the routine care communities (CHCs: n = 403, hospital: n = 180). All the patients participated in this study signed the informed consent. The Ethics Committee of The Luohu Hospital Group and the Eighth Affiliated Hospital, Sun Yat-Sen University in Futian district approved this study.

### Routine and Integrated Care

Integrated care model was implemented in Luohu district, while the original health care was maintained in Futian district. Both two districts are located in the middle of Shenzhen, adjacent to each other, and with similar land areas [21], besides the proportion of civil servants and middle and high-income population in Futian district is higher than that in Luohu district. The economic level and social development of the Futian district was better than that of Luohu [22,23].

In the routine care communities, CHCs were operated and managed directly by individual public hospitals. This mode enabled CHCs to rely on the financial and human resources of their sponsored hospitals. Without a well-organized and coordinated dual referral system, patients preferred to choose high-level hospitals as their first point of contact even for common diseases. The medical insurance mechanism in routine care was the traditional fee-for-service payment in which doctors, hospitals, and CHCs charged separately for each service they provide. According to the national requirements for basic public health services, the CHCs in the routine care carried out screening, diagnosis, treatment and follow-up management of common diseases including hypertension and diabetes mainly through outpatient services [24]. However, the health-care system in routine care remained treatment-oriented, with physicians prescribing when patients went to a clinic rather than offering preventive health care in the early stage of diseases. In addition, a unified electronic information system was not available for different facilities, leading to the fact that patients could receive less integrated and continuous health services.

The integrated care of chronic diseases in Luohu was centred on the health needs of residents, and its bedrock was a strong primary care system, emphasizing disease prevention, health promotion and management, and increment of out-of-hospital care while reducing hospitalization and its costs. Beyond the routine health care, the main infrastructure or approaches for practice of integrated care are summarized as follows. More detailed descriptions of the integrated care in Luohu have been published elsewhere [15,16,17].

(1) Public hospitals and CHCs were vertically integrated into a hospital group which has greater autonomy in personnel, operation, and management, improving the efficiency of resource utilization and administration. Luohu Hospital Group engaged patients to choose CHCs as the first point of contact by strengthening the service capacity of CHCs, with hospitals mainly providing complex care and emergency care for life-threatening situations, as well as some medical care for common diseases and CHCs taking major responsibility for screening, early diagnosis, treatment of common diseases, and rehabilitation, especially for adults with hypertension and diabetes, the elderly and perinatal women. (2) A medical insurance reform called “Global Budget, Balance Retained” was implemented to incentive health providers to diagnose and treat high-risk individuals at the early stage of diseases [25]. (3) Luohu Hospital Group tended to increase the investment of financial and human resources in CHCs to strengthen the capabilities of disease prevention and health management for community residents such as recruiting more young and competent family doctors, strengthening the training of physicians in CHCs, expanding the allocation of drugs and equipping medical devices. (4) The integrated care focused on chronic diseases prevention by family doctor teams which consisted of general practitioners, nurses, and public health physicians. Pharmacists, specialist physicians, or nutritionists would be added when necessary. This multidisciplinary team provided more preventive care, mainly including health education related to lifestyle modification, screening and identifying individuals with hypertension, regular follow-up and management. (5) A new health information system called “Healthy Luohu” has been implemented and shared by all different settings within Luohu district, enabling health providers both in CHCs and hospitals could access patients’ medical records, and provide online management and consultation services. With this application, patients were coordinately referred from CHC to hospitals for complex health care or referred back from hospitals for rehabilitation and primary care conveniently.

### Data Collection

This study was conducted based on the standard protocol with stringent quality control. The quality control group was established to supervise the questionnaire survey, physical examination, laboratory testing, and data entry. Data on demographic characteristics, pharmaceutical expenditure on hypertension and its comorbidities, knowledge, attitude, and practice (KAP) towards cardiovascular diseases, and satisfaction with health care were collected by questionnaires. Standard questionnaires were administered by specially trained physicians and nurses or public health investigators. Each collected questionnaire was double checked by another investigator and the medical expenditure questionnaire was additionally checked by the medical facilities’ billing records database to ensure the accuracy of the information.

Physical examinations provided data on height, weight, and waist circumference. During the measurement, patients were required to take off shoes and wear light clothes. Height, weight, and waist circumference were measured twice. And the means of these measurements were used for analysis. Overnight (at least 8 hours) fasting venous blood samples were collected to measure levels of fasting plasma glucose (FPG), total cholesterol (TC), triglycerides (TG), high-density lipoprotein cholesterol (HDL-C), alanine transferase, aspartate transferase, serum creatinine, and uric acid.

There were 40 questions in the KAP questionnaire, including questions about hypertension and cardiovascular disease-related knowledge, attitudes, and behaviour changes. Each question was 2.5 points. For multiple-choice questions, 2.5/n_options_ points were awarded for each correct choice of an answer or successful avoidance of an incorrect answer. The high KAP scores represented the high level of the patient’s knowledge, attitude, and behaviour towards cardiovascular diseases. Patients’ satisfaction with health services included six aspects, which were assessed by a 5-point Likert scale (Very dissatisfied, Dissatisfied, Neutral, Satisfied, and Very satisfied). The satisfaction rate referred to the proportion of patients who chose “Satisfied” or “Very Satisfied”.

### Blood pressure (BP) measurement

Participants were required to sit and rest for at least 5 minutes for BP measurement with electronic sphygmomanometers. They were asked to avoid exercise, smoking, and drinking caffeinated beverages 1 hour before the measurement. BP was measured 3 times, and the mean of the 3 BP measurements was used for analysis.

### Definitions

Hypertension was defined as SBP ≥ 140 mmHg and/or DBP ≥ 90 mmHg and/or using antihypertensive medication within two weeks prior to the baseline examination [20]. Diabetes was defined as FPG ≥126 mg/dl (7.0 mmol/L) or current treatment with oral hypoglycemic agents or insulin injection [26]. Dyslipidemia was defined as self-reported current treatment with cholesterol-lowering medication or having 1 or more of the following: TC ≥ 240 mg/dl (6.22 mmol/L), TG ≥ 200 mg/dl (2.26 mmol/L), HDL-C ≤ 40 mg/dl (1.04 mmol/L), or low-density lipoprotein cholesterol ≥ 160 mg/dl (4.14 mmol/L) [27]. Comorbidity status was defined as in addition to hypertension, the participants also suffered from diabetes, dyslipidemia, chronic respiratory or kidney diseases.

The standardized management of hypertensive patients was defined as patients receiving standard treatment by clinicians following the “National Standards for Basic Public Health Services 2017”, at least 4 follow-up visits per year and 1 comprehensive health check-up [28]. Hypertension control refers to SBP < 140 mmHg and DBP < 90 mmHg after drug therapy or nonpharmacological management. Medication compliance was divided into non-compliance, occasional compliance, and complete compliance according to whether they take the medicine regularly as prescribed by doctors.

### Statistical Analysis

Basic characteristics were presented according to routine care and integrated care. Continuous variables were presented as median (25^th^–75^th^ percentile) and compared by Wilcoxon test. Categorical variables were presented as numbers (percentages) and compared by Chi-square test followed by Bonferroni post-hoc test. KAP scores were presented as means with standard deviations and were tested by T-test. The age-standardized control rate of hypertension was calculated by direct standardization and compared by Z-test. The pharmaceutical expenditure on hypertension and its comorbidities was classified into three groups: <Ұ125/month, Ұ125–300/month, and ≥300/month, and then compared by Chi-square test. The pharmaceutical expenditure also described with means ± standard deviations. A two-tailed *P* < 0.05 was considered statistically significant. All statistical analyses were performed using SAS software version 9.4 (SAS Institute Inc, Cary, NC, USA).

## Results

### Characteristics of study participants

The current study included 583 participants from the routine care communities and 1353 participants from the integrated care communities. The characteristics of these hypertensive patients by different care are presented in Table 1. Patients from the integrated care communities were slightly younger than those from routine care communities (integrated care: 57.0 years, routine care: 58.5 years, *P* = 0.006). 57.21% of patients from integrated care communities had the household income per capita of ≤Ұ5000/month, 14.42% of patients had the household income per capita of >Ұ10000/month, while those in routine care communities were 61.79% and 9.17%, respectively (*P* = 0.015). Compared with routine care, a higher proportion of patients in integrated care communities chose CHCs as the first point of contact for medical service (integrated care: 85.64%, routine care: 79.38%, *P* < 0.0001). However, among patients from the routine care communities, the coverage rate of medical insurance was higher (integrated care: 74.13%, routine care: 81.14%), and a higher proportion of them was first-grade medical insurance (integrated care: 56.69%, routine care: 61.41%) (*P* < 0.0001).

**Table 1:** Comparison of basic and clinical characteristics of participants with hypertension from the integrated care and the routine care communities.


CHARACTERISTICS	ROUTINE CARE COMMUNITIES	INTEGRATED CARE COMMUNITIES	χ^2^/*Z*	*P*

**Sociodemographic**				

Men, n (%)	346 (59.35)	746 (55.14)	2.939	0.087

Age, year	58.5 (50–66)	57.0 (49–64)	2.749	**0.006**

Education, n (%)			1.432	0.489

Middle school or below	245 (42.39)	558 (41.27)		

High school	192 (33.22)	486 (35.95)		

College degree or above	141 (24.39)	308 (22.78)		

Married/Cohabitating, n (%)	528 (90.88)	1251 (92.53)	1.512	0.219

Occupation, n (%)			1.927	0.588

Worker	36 (6.02)	106 (7.84)		

Salaried employee	144 (24.78)	336 (24.85)		

Self-employed	45 (7.75)	111 (8.21)		

Retiree or other	356 (61.27)	799 (59.10)		

Current drinker, n (%)	154 (26.46)	344 (25.44)	0.220	0.639

Current smoker, n (%)	191 (32.76)	394 (29.12)	2.562	0.110

Household income per capita, Ұ/month, n (%)			8.344	**0.015**

≤5000	283 (61.79)	706 (57.21)		

5001–10000	133 (29.04)	350 (28.36)		

>10000	42 (9.17)	178 (14.42)		

Medical insurance, n (%)			22.71	**<0.0001**

First grade	358 (61.41)	767 (56.69)		

Second grade	83 (14.24)	192 (14.19)		

Third grade	32 (5.49)	44 (3.25)		

Self-paying or other	110 (18.87)	350 (25.87)		

The usual source of care, n (%)			28.154	**<0.0001**

CHCs	458 (79.38)	1157 (85.64)		

Public hospital	76 (13.17)	161 (11.92)		

Private hospital or other	43 (7.45)	33 (2.44)		

**Clinical**				

Comorbidity status, n (%)	261 (44.77)	569 (42.05)	1.225	0.268

BMI, kg/m^2^	25.8 (23.6–27.9)	25.6 (23.7–27.8)	0.364	0.716

WC, cm	90.6 (84.4–96.5)	90.0 (83.6–95.7)	1.948	0.051

SBP, mmHg	136.0 (127.0–146.0)	137.0 (127.3–147.3)	–0.970	0.332

DBP, mmHg	82.0 (77.3–97.3)	84.3 (77.3–91.0)	–1.483	0.138

FPG, mmol/L	5.9 (5.4–6.7)	5.6 (5.2–6.3)	5.407	**<0.0001**

With diabetes	7.3 (6.2–8.9)	7.2 (6.4–8.2)	0.914	0.361

Without diabetes	5.7 (5.3–6.2)	5.4 (5.1–5.9)	5.735	**<0.0001**

Scr, umol/L	68.0 (57.5–80.0)	67.0 (56.0–78.0)	1.618	0.106

UA, umol/L	352 (300–419)	354 (298–422)	–0.209	0.835

TC, mmol/L	5.1 (4.4–5.9)	5.0 (4.3–5.7)	1.583	0.114

TG, mmol/L	1.4 (1.0–2.1)	1.4 (1.0–2.0)	1.117	0.264

HDL-C, mmol/L	1.2 (1.0–1.4)	1.2 (1.0–1.4)	0.386	0.699

ALT, IU/L	18.5 (13.0–26.0)	20.0 (15.0–29.0)	–3.798	**0.0001**

AST, IU/L	22.0 (19.0–26.0)	22.0 (19.0–26.0)	–0.694	0.488


*Notes*: **a)** *P* was calculated by chi-square test (categorical variables) and Wilcoxon test (continuous variables); **b)** Continuous variables are presented as medians (25^th^–75^th^ percentile); **c)** CHCs: community health centre; ALT: alanine transferase; AST: aspartate transferase; BMI: body mass index; DBP: diastolic blood pressure; FPG: fasting plasma glucose; HDL-C: high density lipoprotein cholesterol; SBP: systolic blood pressure; Scr: serum creatinine; TC: total cholesterol; TG: triglyceride; UA: uric acid; WC: waist circumference.

### Hypertension management and control

Among these hypertensive patients, the standardized management population accounted for 14.07% in the routine care communities and 45.75% in the integrated care communities (*P* < 0.0001) (Table 2). Among these hypertensive patients with standardized management, 2.15% of the patients from integrated care communities were non-compliant (routine care: 12.00%), and 88.91% of the patients were completely compliant (routine care: 85.33%), with *P* value < 0.0001.

**Table 2:** Hypertension management and control in the integrated care and the routine care communities.


HYPERTENSION MANAGEMENT AND CONTROL		ROUTINE CARE COMMUNITIES	INTEGRATED CARE COMMUNITIES	χ^2^/*Z*	*P*

**Hypertension management**					

Standardized management, n (%)		82 (14.07)	619 (45.75)	177.09	**<0.0001**

Medication compliance, n (%)					

Participants with standardized management	Non-compliance*	9 (12.00)	13 (2.15)	23.249	**<0.0001**

	Occasional compliance	2 (2.67)	54 (8.94)		

	Complete compliance	64 (85.33)	537 (88.91)		

Participants without standardized management	Non-compliance	38 (7.98)	44 (6.32)	2.461	0.292

	Occasional compliance	54 (11.34)	96 (13.79)		

	Complete compliance	384 (80.67)	556 (79.89)		

**Mean BP level and hypertension control rate**					

All participants	SBP, mmHg	137.3 ± 16.0	138.0 ± 15.6	–1.194	0.232

	DBP, mmHg	83.8 ± 11.2	84.3± 10.7	–1.483	0.138

	Age-standardized control rate, % (95% CI)	52.65 (46.54–58.77)	50.30 (46.49–54.10)	0.650	0.518

Comorbidity status					

Yes	SBP, mmHg	137.0 ± 15.5	136.5 ± 15.6	0.233	0.816

	DBP, mmHg	82.2 ± 10.8	82.3 ± 10.7	–0.043	0.966

	Age-standardized control rate, % (95% CI)	56.62 (46.30–66.94)	52.76 (46.69–58.82)	0.640	0.521

No	SBP, mmHg	137.5 ± 16.4	139.1 ± 15.5	–1.641	0.101

	DBP, mmHg	84.9 ± 11.4	85.7 ± 10.5	–1.697	0.090

	Age-standardized control rate, % (95% CI)	50.99 (43.04–58.94)	48.81 (43.85–53.76)	0.460	0.645

Gender					

Men	SBP, mmHg	136.6 ± 15.6	137.8 ± 14.9	–1.220	0.223

	DBP, mmHg	85.1 ± 11.4	86.6 ± 10.1	–2.484	**0.013**

	Age-standardized control rate, % (95% CI)	50.89 (43.07–58.71)	47.69 (42.58–52.80)	0.680	0.496

Women	SBP, mmHg	138.2 ± 16.5	138.2 ± 16.3	–0.345	0.730

	DBP, mmHg	81.9 ± 10.8	81.4 ± 10.7	0.140	0.889

	Age-standardized control rate, % (95% CI)	55.37 (44.61–66.13)	54.36 (48.29–60.44)	0.160	0.873


*Notes*: **a)** SBP: systolic blood pressure; DBP: diastolic blood pressure; 95% CI: 95% confidence interval; **b)** Variables are mean ± standard deviation unless otherwise indicated; **c)** *P* for hypertension management was calculated by chi-square test, *P* for SBP/DBP and age-standardized control rate of hypertension was calculated by Wilcoxon test and z-test; **d)** * *P* < 0.0001, Bonferroni-adjusted inspection level of α = 0.0167.

The mean SBP level of patients with hypertension from the integrated care communities was 138.0 ± 15.6 mmHg, which was similar to that of 137.3 ± 16.0 mmHg from the routine care communities (*P* = 0.232). The age-standardized control rate of hypertension was not statistically significant between the integrated and routine care communities, with rates of 50.30% (95% CI: 46.49–54.10) and 52.65% (95% CI: 46.54–58.77) (*P* = 0.518), respectively. Similar results were also obtained when stratified by comorbidity status and gender.

### Pharmaceutical expenditure on hypertension and its comorbidities

**Figure 2:**
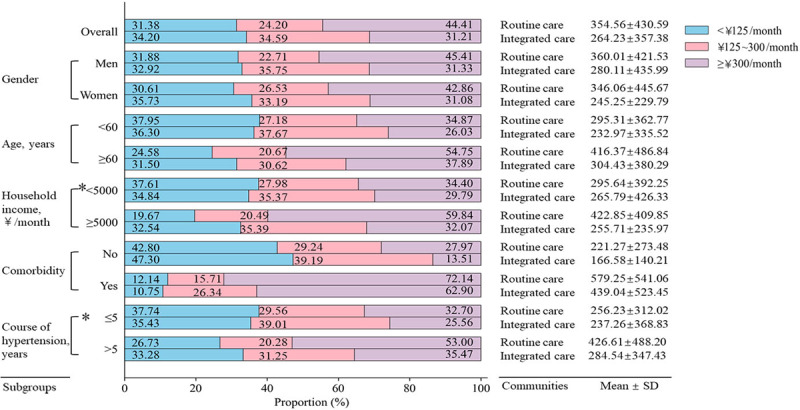
The monthly pharmaceutical expenditure on hypertension and its comorbidities per capita in the integrated care and the routine care communities. *P* was calculated by Chi-square test, and all *P* < 0.05, except for *.

Figure 2 shows monthly pharmaceutical expenditure on hypertension and its comorbidities of patients with different characteristics in routine care and integrated care communities. The proportion of patients with pharmaceutical expenditure ≥Ұ300/month from integrated care communities was lower than those from routine care communities (31.21% versus 44.41%, *P* < 0.0001). The average pharmaceutical expenditure in the integrated care communities was only Ұ264.23 ± 357.38/month/person, versus Ұ354.56 ± 430.59/month/person in the routine care communities. For patients aged over 60 years, the monthly expenditure on pharmaceuticals in the integrated care communities was also substantially lower than that in the routine care (integrated care: Ұ304.43 ± 380.29/month/person, routine care: Ұ416.37 ± 486.84/month/person). Similar findings were also found among patients whose household income was >Ұ5000/month (integrated care: Ұ255.71 ± 235.97/month/person, routine care: Ұ422.85 ± 409.85/month/person), or whose course of hypertension was >5 years (integrated care: Ұ284.54 ± 347.43/month/person, routine care: Ұ426.61 ± 488.20/month/person).

### KAP scores towards cardiovascular diseases

The total KAP scores towards cardiovascular diseases in the integrated care and routine care communities were 73.48 ± 11.54 and 68.89 ± 15.51 (*P* < 0.0001), indicating that the level of cardiovascular health-related knowledge in the integrated care communities was higher than routine care communities (Table 3). The scores of knowledge (integrated care: 32.10, routine care: 29.83), attitude (integrated care: 21.76, routine care: 20.33), and practice (integrated care: 19.61, routine care: 18.73) of patients from the integrated care communities were also slightly higher than that from the routine care communities (all *P* values < 0.0001). Subgroup analysis stratified by standardized management verified the robustness of the results.

**Table 3:** The Knowledge, attitude, and practice scores towards cardiovascular diseases of participants with hypertension from the integrated care and the routine care communities.


KAP scores	Routine care communities	Integrated care communities	t values	***P***

**All participants**				

Knowledge score	29.83 ± 8.92	32.10 ± 6.35	–5.580	**<0.0001**

Attitude score	20.33 ± 5.56	21.76 ± 4.36	–5.530	**<0.0001**

Practice score	18.73 ± 4.53	19.61 ± 4.37	–4.000	**<0.0001**

Total score	68.89 ± 15.51	73.48 ± 11.54	–6.430	**<0.0001**

**Participants with standardized management**				

Knowledge score	29.32 ± 9.25	32.64 ± 5.58	–3.180	**0.002**

Attitude score	20.55 ± 5.58	22.24 ± 3.84	–2.660	**0.009**

Practice score	20.03 ± 3.98	20.99 ± 3.58	–2.080	**0.040**

Total score	69.90 ± 15.46	75.88 ± 9.52	–3.420	**0.001**

**Participants without standardized management**				

Knowledge score	29.91 ± 8.88	31.65 ± 6.90	–3.690	**0.0002**

Attitude score	20.29 ± 5.56	21.36 ± 4.71	–3.520	**0.0004**

Practice score	18.51 ± 4.58	18.45 ± 4.63	0.230	0.814

Total score	68.72 ± 15.52	71.47 ± 12.66	–3.280	**0.001**


*Notes*: **a)** *P* was calculated by T test; **b)** Variables are mean ± standard deviation; **c)** KAP: knowledge, attitude, and practice.

### Patients’ satisfaction with health service delivery

**Figure 3:**
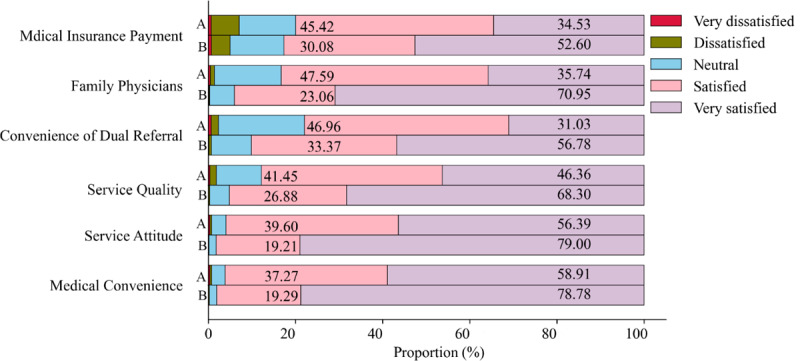
Patients’ satisfaction with health service delivery in the routine care **(A)** and the integrated care communities **(B)**. *P* was calculated by χ^2^ test to compare the difference of 5-point satisfaction between routine care (A) and integrated care communities (B). And all *P* values were < 0.0001.

Figure 3 shows the satisfaction with health service delivery in routine care and integrated care communities. Compared with routine care, patients from integrated care communities were more satisfied with medical convenience, service attitude, dual referral convenience, service quality, family physicians, and medical insurance payment. In terms of the convenience of two-way referral, the overall satisfaction rate in routine care was 77.99% (Very Satisfaction: 31.03%+Satisfaction: 46.96%), while that in the integrated care was 90.15% (56.78%+ 33.37%) (*P* < 0.0001).

## Discussion

The current study in two districts of Shenzhen investigated the short-term impact of the integrated care model on hypertension management, including hypertension control rate, pharmaceutical expenditure on hypertension and its comorbidities, KAP towards cardiovascular diseases, and health service satisfaction among hypertensive patients. While with a similar control rate of hypertension between the integrated and routine care communities, the implementation of the integrated care model has substantially increased the rate of standardized hypertension management and improved the knowledge, attitude, and behaviour changes of hypertensive patients. Moreover, individual patient’s medical expenditure in the integrated care communities was less than that in the routine care communities, which was mainly driven by medical insurance reforming. As a result, a higher proportion of patients in the integrated care communities were satisfied with health services in the CHCs.

The increment in KAP scores represented the improvement in health literacy among hypertensive patients. One study illustrated that the health literacy level of residents in the Luohu district in 2018 increased by 21.94% compared to 2016 [29]. Similar to the Luohu model, the integrated care model developed in other countries and regions also found positive changes in patients’ knowledge, attitude, and behaviour. “Healthy kinzigtal” was an integrated care model launched in Germany in 2004 [30]. After 9 years of operation of this model, 24% of patients lived healthier than before [31]. These results may be due to the close contact between patients and medical staff, which could help improve patients’ awareness and skills of self-health management [32]. One of the important measures of the Luohu model was to build the service capacity of CHCs and use the family physician teams as the starting point for health services [33]. The integrated care in Luohu encouraged patients to sign up health services with family doctors, thereby enhancing the interaction between patients and medical staff. The high rate of standardized management may also be the result of the integrated care model by strengthening the interaction between patients and family doctors.

The reduction of patients’ pharmaceutical expenditure reflected that the early practice of the integrated care in Luohu may improve the efficiency of medical health services. The major move of the Luohu model is the reform of the medical insurance payment, in addition to a mechanism in favour of career development for medical personnel in CHCs. In which there was a strong economic incentive to encourage hospitals to take the initiative to facilitate health promotion, early screening, prevention and management of adults with high risks at the community level rather than treating patients with the late stage in hospitals. The goals of Luohu Hospital Group have changed to achieve less incident chronic diseases, less hospitalizations and less medical payment by residents. Through a global budget mechanism, the Luohu Hospital Group has achieved a new finance balance by providing higher-quality CHCs health services with a less residents’ expensive medical spending in hospitals and more budget surplus for Luohu Hospital Group itself [15]. Our findings are consistent with other studies investigating the effects of global budget [34]. Maryland’s global hospital budgets also reduced the expenditure of medical care without having adverse impacts on hospital finances [34]. In 2016, a study in China investigated hospitals with different payment methods and found that the global budget can not only control the growth of medical care costs but also promote the improvement of medical quality [35]. Therefore, the global budget unified the interests of medical facilities, physicians, and patients.

A much better patients’ satisfaction with healthcare services indicated the improvement of the quality of medical services in the Luohu model. The integrated care in Luohu has improved patients’ satisfaction with medical convenience, service quality, service attitude, and medical insurance payment. This result is similar to the findings of the previous survey on patient satisfaction with health service delivery in the Luohu district, which found that more than 90% of patients gave good or very good feedback on the healthcare service they obtained [36]. According to the annual satisfaction of Shenzhen municipal health commission, in 2018, the satisfaction survey covered 1.12 million inpatients and outpatients via telephone and found that residents’ satisfaction with Luohu Hospital Group was higher than that of routine care communities [37]. The improvement in satisfaction was the inevitable result of medical facilities providing better health services and lower medical expenditure [38,39].

The current study has important public health implications. First, this study identified 3 years of integrated care could improve the standardized management rate of hypertension and patient compliance. Second, the early practice of the integrated care to build the capacity of CHCs could effectively improve the service quality of primary medical facilities, and promote residents to actively choose CHCs as the usual source of medical care, which play an important role in a hierarchical medical system. Finally, the global budget could effectively control and reduce the medical expenditure of patients with hypertension.

Some limitations of this analysis should be noted. First, with the lack of longitudinal data, it was unable to assess the incident risks of cardiovascular disease among hypertensive patients in the integrated or routine care communities. Second, only patients with hypertension were included in this study thus no impact of the integrated care on other chronic diseases were addressed. Future researches using a real-world design with larger samples on hypertension management and cardiovascular diseases prevention in integrated care communities were warranted to better examine and improve the integrated care in Luohu.

## Conclusion

The early practice of the integrated care in Luohu substantially strengthened the hypertension management and improved the efficiency and quality of medical services. The improvement of hypertension control rate may require longer observation or larger data assessment. The integrated care in Luohu should further be emphasized with the individualized hypertension management to improve the service quality, hypertension control rate and reduce cardiovascular diseases in population.

## Abbreviations

CHCs = Community health centres

KAP = Knowledge, attitude, and practice

BP = Blood pressure

SBP = Systolic blood pressure

DBP = Diastolic blood pressure

ALT = Alanine transferase

AST: = Aspartate transferase

BMI = Body mass index

FPG = Fasting plasma glucose

HDL-C = High density lipoprotein cholesterol

Scr = Serum creatinine

TC = Total cholesterol

TG = Triglyceride

UA = Uric acid

WC = Waist circumference
